# Ig-Like Transcript 2 (ILT2) Blockade and Lenalidomide Restore NK Cell Function in Chronic Lymphocytic Leukemia

**DOI:** 10.3389/fimmu.2018.02917

**Published:** 2018-12-11

**Authors:** Mónica Villa-Álvarez, Christian Sordo-Bahamonde, Seila Lorenzo-Herrero, Ana P. Gonzalez-Rodriguez, Angel R. Payer, Esther Gonzalez-Garcia, María C. Villa-Álvarez, Alejandro López-Soto, Segundo Gonzalez

**Affiliations:** ^1^Department of Functional Biology, University of Oviedo, Oviedo, Spain; ^2^University Institute of Oncology (IUOPA), University of Oviedo, Oviedo, Spain; ^3^Instituto de Investigación Sanitaria del Principado de Asturias, Oviedo, Spain; ^4^Department of Hematology, Hospital Universitario Central de Asturias, Oviedo, Spain; ^5^Department of Hematology, Hospital de Cabueñes, Gijón, Spain; ^6^Department of Emergency Medicine, Hospital Vital Álvarez Buylla, Mieres, Spain

**Keywords:** chronic lymphocytic leukemia, NK cells, ILT2, lenalidomide, IL-2, checkpoint

## Abstract

One of the cardinal features of chronic lymphocytic leukemia (CLL) is its association with a profound immunosuppression. NK cell function is markedly impaired in CLL patients, who show a significant dysregulation of the expression of activating and inhibitory receptors. Here, we analyzed the role of the novel inhibitory receptor Ig-like transcript 2 (ILT2, also termed LIR-1, LILRB1) in the regulation of NK cells in CLL. Our results show that ILT2 expression was significantly decreased on leukemic cells and increased on NK cells of CLL patients, particularly in those with advanced disease and with bad prognostic features, such as those carrying chromosome del(11q). The immunomodulatory drug lenalidomide may regulate the expression of ILT2 and its ligands in CLL since it significantly increased the expression of ILT2 and partially reestablished the expression of its ligands on leukemic cells. Furthermore, lenalidomide significantly increased the activation and proliferation of NK cells, which was strongly enhanced by ILT2 blockade. Combining ILT2 blockade and lenalidomide activated NK cell cytotoxicity resulting in increased elimination of leukemic cells from CLL patients. Overall, we describe herein the role of an inhibitory receptor involved in the suppression of NK cell activity in CLL, which is restored by ILT2 blockade in combination with lenalidomide, suggesting that it may be an interesting therapeutic strategy to be explored in this disease.

## Introduction

Chronic lymphocytic leukemia (CLL) is a lymphoproliferative malignancy characterized by the accumulation of clonal mature B cells in lymphoid organs, bone marrow, and peripheral blood. CLL patients display significant clinical heterogeneity, ranging from patients with indolent disease to patients with advanced stage disease, which require chemotherapy treatment. This clinical heterogeneity is associated with a heterogeneous array of chromosomic, genetic, and molecular defects (1). Thus, patients harboring chromosome del(17p) or del(11q) have been associated with a poor clinical outcome (2–4), but CLL patients with del(13q) have been associated with a more favorable prognosis (5, 6).

Natural Killer (NK) cells are innate immune cells that play a key role in the immunosurveillance of hematopoietic malignancies. In CLL, NK cells are increased at diagnosis and early stage disease (7, 8). However, NK cell function is markedly impaired in advanced patients showing a profound deficiency of cytotoxic molecules and activating receptors (9–11). Particularly, the over-expression on NK cells of inhibitory receptors and its ligands may contribute to disease pathogenesis and resistance to immunotherapy (12, 13). Interestingly, targeting these inhibitory checkpoints has achieved noteworthy benefit in cancer patients by restoring an effective antitumor response (14, 15).

ILT2 (also named CD85j, LILRB1, or LIR-1) is an inhibitory receptor expressed by T cells, B cells, NK cells, and other immune cells (16–18). ILT2 ligands are both classical (HLA-A, -B, and -C) and non-classical MHC class I molecules (19–21). Specifically, ILT2 binds to HLA-G with a three- to four-fold higher affinity than to classical MHC class I molecules (21). The interaction of ILT2 with its ligands impairs the function and effector activity of NK cells (17, 21). Further, ILT2 inhibits the polarization of NK cell lytic granules and the microtubule organizing center (MTOC) and the accumulation of filamentous actin (F-actin) at the area of contact inhibiting intracellular calcium mobilization and IFN-γ polarized production by NK cells (22).

Immunomodulatory drugs, such as lenalidomide, have changed the therapeutic landscape in CLL showing that targeting the immune system represents an efficient therapeutic strategy in this disease (23–27). Lenalidomide is unable to induce direct apoptosis of leukemic CLL cells (25), but it regulates critical pro-survival and angiogenic cytokines and promotes the activation of T cells (28, 29). Moreover, it also increases NK cell proliferation, which correlates with clinical response (29–31). Significantly, the proliferation and activation of NK cells in CLL is mediated, at least in part, by the production of IL-2 by CD4 T cells (32). Lenalidomide also enhances NK cell-mediated natural and Antibody-Dependent-Cell-Mediated Cytotoxicity (ADCC) against leukemic cells of CLL patients (32–34).

We recently reported a crucial role of ILT2 in the impairment of T cell function in CLL (35). In the present work, we show that ILT2 is also involved in the suppression of NK cells in CLL and we report that the combination of ILT2 blockade with lenalidomide restores NK cell function favoring the elimination of leukemic cells.

## Patients, Materials, and Methods

### Patients

Sixty consecutive non-treated CLL patients from the Hospital Universitario Central de Asturias fulfilling the diagnostic criteria for CLL (Table 1), and 25 age-matched healthy donors (mean age 59.5 years) were studied. This study was approved by the Ethics Committee of our institution (Comité de Ética de la Investigación del Principado de Asturias, 19042016) and informed consent according to the Declaration of Helsinki was obtained from all patients and controls. Both clinical and immunological characteristics of patients were analyzed when patients were enrolled in this study. Clinical and laboratory evaluation at visit included history and physical examination, standard clinical laboratory evaluation, evaluation for ZAP-70 by flow cytometry (20% cut-off), characterization of CD38 expression by flow cytometry (30% cut-off); and standard metaphase karyotype. Karyotype was categorized as complex, single abnormality or diploid. FISH analysis for 17p deletion, 11q deletion, trisomy 12, and chromosome 13q deletion was also performed. A total of 200 cells in interphase were analyzed for each probe. Positive patient cases were those with 5% or more cells with the abnormality. IGHV mutation status was characterized by direct sequencing method, and patients were categorized as unmutated (IGHV 98% germline homology) or mutated (98% homology).

**Table 1:** Clinical characteristics of CLL patients.

	**Patients**	
	**60**	**%**
Age (years, mean)	67.4
**SEX**		
Male	31	51.6
Female	29	49.3
**BINET STAGE**		
A	44	73.3
B	7	11.6
C	9	15
**RAI STAGE**		
0	27	45
I	16	26.6
II	6	10
III-IV	11	18.3
**ECOG PERFORMANCE STATUS**		
0	47	78.3
1	9	15
2	4	6.6
**CYTOGENETIC ABNORMALITIES (FISH) (*****n*** **=** **54)**		
del(17p)	4	7.4
del(11q)	5	9.2
del(13q)	37	68.5
Trisomy 12	8	14.8
**METAPHASE KARYOTYPE (*****n*** **=** **44)**		
Complex	6	13.6
Single abnormality	14	31.8
Diploid	24	54.5
***IGHV*** **MUTATION STATUS (*****n*** **=** **47)**		
Unmutated	13	27.6
Mutated	31	65.9
Discordant	3	6.3
**CD38 EXPRESSION (*****n*** **=** **54)**		
Positive (≥30%)	10	18.5
**ZAP-70 (*****n*** **=** **38)**		
Flow positive (≥20%)	8	21
Progressive disease	34	56.6
Stable disease	26	43.3

### Antibodies and Materials

Recombinant human interleukin 2 (IL-2; Peprotech), lenalidomide (reconstituted in DMSO; Selleckchem), sCD40L (Peprotech), HP-F1 anti-ILT2/CD85j blocking antibody (36, 37) (IgG1) provided by Miguel López-Botet (Universitat Pompeu Fabra, Spain) and an irrelevant IgG1 antibody (provided by JR de los Toyos-González, Universidad de Oviedo, Spain) were used in this study.

### Flow Cytometry

For analyzing the expression of ILT2 and its ligands in different lymphocyte subsets, 1 × 10^6^ PBMCs obtained from CLL patients and healthy donors were incubated with the corresponding antibodies at room temperature for 20 min. Anti-CD3-FITC, anti-CD56-APC, and anti-CD19-APC antibodies (all from Immunostep), anti-CD3-PECy7 and anti-CD56-PECy7 antibodies (eBioscience), and appropriate isotype-matched controls were used to define the immune subsets. Anti-ILT2-PE (clone HP-F1, eBioscience), anti-HLA-F rabbit antibody (Abgent), anti-HLA-G-PE (clone 87G Biolegend), anti-HLA-E-PE (clone 3D12 Biolegend), and a pan-anti-HLA-I antibody (clone W6/32) were used to measure the expression of ILT2 and its ligands in NK cells and B cells. NK cells were defined as CD3^−^CD56^+^, and B cells as CD19^+^. Cells were analyzed on a BD Biosciences FACS Canto II cytometer (Beckton Dickinson).

### NK Cell Activation

Peripheral blood mononuclear cells (PBMCs) obtained from CLL patients were cultured in the presence of anti-ILT2 blocking antibody (HP-F1; 10 μg/mL) or irrelevant mouse IgG1, and lenalidomide (1 μM) and IL-2 (50 U/ml) for 7 days. The expression of CD69, CD25, CD137, NKG2D, and DNAM-I on NK cells (defined as CD3^−^CD56^+^) was evaluated by flow cytometry analysis using specific antibodies; anti-CD69-FITC (Immunostep), anti-CD25-PE (Biolegend), anti-CD137-PE (Biolegend), anti-NKG2D-PE (Miltenyi), and anti-DNAM-I-PE (Biolegend).

### Cell Proliferation

PBMCs obtained from CLL patients were labeled with 1 μM 5,6-carboxyfluorescein diacetate succinimidyl ester (CFSE; Sigma-Aldrich) and cultured in the presence or absence of the anti-ILT2 blocking antibody (HP-F1) and lenalidomide (1 μM) for 14 days. Fresh lenalidomide was added to cell culture every 72 h. Then, cells were stained with specific antibodies for NK cells and leukemia cells; and CFSE staining of different cell populations was analyzed by flow cytometry as we previously reported (32).

### Leukemia Depletion Assay and Apoptosis

PBMCs from CLL patients were cultured in the presence or absence of anti-ILT2 blocking antibody (HP-F1) and lenalidomide for 7 days. Then, samples were stained with anti-CD19-FITC; and PKH26 reference microbeads (Sigma) were added to the samples according to manufacturer's instructions. Flow cytometry analysis was performed by displaying 5,000 microbeads for each tube. CD19^+^ B-cell populations were gated, and the absolute counts of B cells were determined. The B cell numbers obtained with the antibody-untreated samples were set as 100% cells (equal 0% cell depletion). PBMCs from 11 CLL patients were cultured in the presence of the anti-ILT2 blocking antibody (HP-F1) and lenalidomide for 7 days, and apoptosis was evaluated using the Annexin V Assay and by flow cytometry analysis.

### NK Cell Cytotoxicity and Perforin Production by NK Cells

In order to determine NK cell cytotoxic capacity, a calcein-based assay was performed. Briefly, effector cells (PBMCs from 5 patients with CLL) were cultured in the presence of anti-ILT2 blocking antibody (10 μg/mL) or mouse IgG1 (10 μg/mL) and lenalidomide (1 μM) for 7 days. Afterwards, K562 leukemia cells (ATCC) were labeled with calcein-AM (Invitrogen) following the manufacturers' protocol and used as target cells. Specifically, 2 × 10^4^ K562 cells were co-cultured with effector cells at an 10:1 (E:T) ratio for 4 h in a 96-well plates. Calcein release was measured on a Varioskan™ LUX multimode microplate reader. Intracellular perforin expression was evaluated on NK cells from PBMCs obtained from patients with CLL upon treatment with lenalidomide (1 μM), ILT2-blocking antibody (10 μg/ml) or irrelevant mouse IgG1 for a week. Then, cells were washed and incubated with PMA (50 ng/ml), ionomycin (1 μM) and brefeldin A (1 μM) for 4 h. Afterwards, cells were stained with anti-CD3 and anti-CD56 antibodies and permeabilized using BD Cytofix/Cytoperm^TM^ solution kit. Perforin intracellular staining was evaluated in NK cells (CD3^−^CD56^+^) using a PerCP/Cy5.5 conjugated anti-human perforin antibody (Biolegend) following manufacturer's recommendations by flow cytometry analysis.

### Statistics

The relationship between continuous and categorical prognostic variables was evaluated by Mann–Whitney *U*-test, and Wilcoxon Matched-Pairs Signed Ranks test was used for intra-group comparisons.

## Results

### ILT2 Expression on NK Cells Is Increased in Patients With CLL

**Figure 1:**
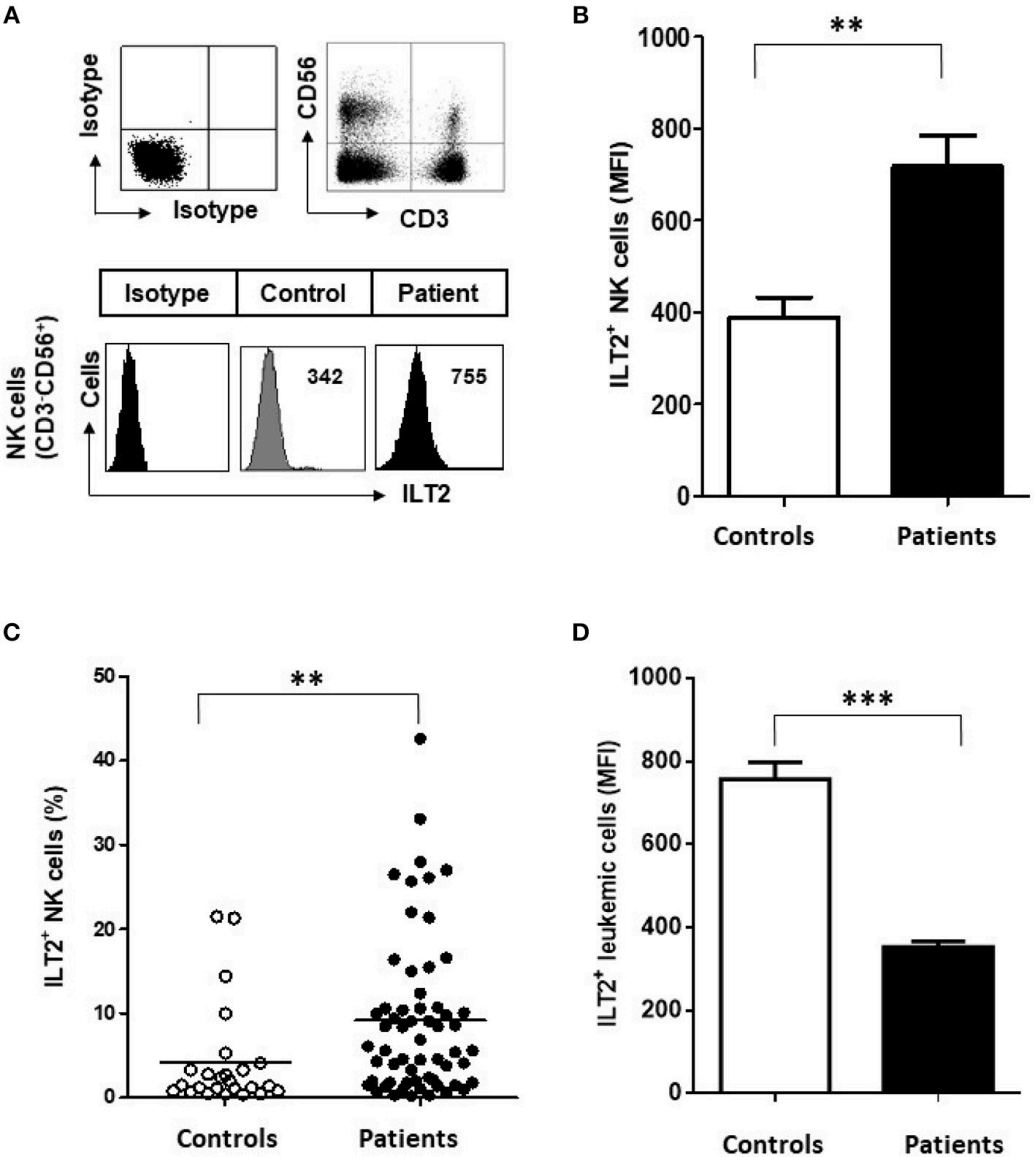
Surface ILT2 expression is increased on NK cells of CLL patients. **(A)** The expression of ILT2 was analyzed in PBMCs from 60 CLL patients and 25 healthy donors by flow cytometry. The histogram shows the ILT2 expression on NK cells (CD3^−^CD56^+^) from a representative healthy donor and a patient with CLL. **(B)** The comparison between the MFI ±SEM of ILT2 surface expression on NK cells from healthy controls (*n* = 25) and patients with CLL (*n* = 60) is shown. **(C)** The comparison between the percentage of ILT2^+^ NK cells from healthy controls and patients is shown. Horizontal bars represent the mean ± SEM. **(D)** The comparison between the MFI ± SEM of ILT2 surface expression on leukemic cells and B cells from healthy controls is shown. SEM, Standard Error of the Mean; Mann-Whitney *U*-test; ^**^P < 0.01, ^***^P < 0.001).

The expression of ILT2 on NK cells was analyzed in 60 patients with CLL and 25 healthy controls by flow cytometry (Figure 1A). Compared with healthy controls, the level of ILT2 expression on NK cells (Mean Fluorescence Intensity (MFI): 389.2 ± 217.8 vs. 717.9 ± 517.8, *P* < 0.01; Figure 1B) and the percentage of ILT2^+^ NK cells (4.2 ± 6 vs. 8.6 ± 9.1, *P* < 0.01; Figure 1C) were significantly increased in CLL patients. Contrarily, and in agreement with our previous report (35), ILT2 expression was significantly decreased on leukemic cells (Figure 1D). Of note, ILT2 expression on B cells from healthy donors was not altered by the treatment with the B cell activator molecule sCD40L, suggesting that ILT2 expression on B cells is not modulated by the activation status (data not shown).

**Figure 2:**
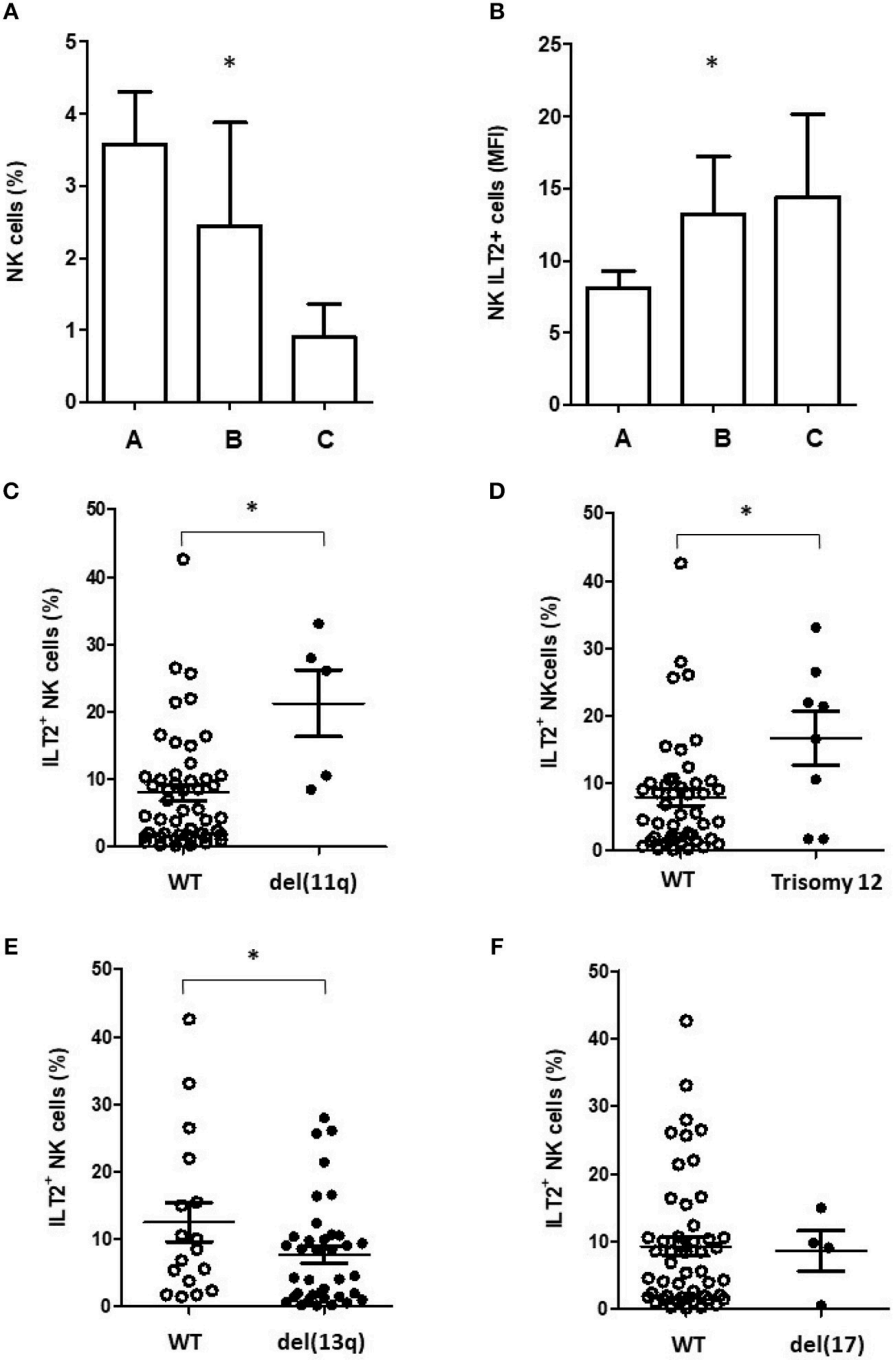
ILT2 expression on NK cells associates with bad prognostic features of CLL patients. Histograms show the comparison of NK cells **(A)** and ILT2^+^ NK cells **(B)** percentages among CLL patients stratified by the Binet stage. Comparison of the percentage of ILT2^+^ NK cells in CLL patients stratified by the presence of chromosome del(11q) **(C)**, trisomy 12 **(D)**, del(13q) **(E)**, and del(17) **(F)**. Horizontal bars represent the mean ± SEM. SEM, Standard Error of the Mean; Mann-Whitney *U*-test; ^*^P < 0.05.

Clinical analysis show that the percentage of NK cells was significantly decreased in patients with advanced stage of Binet system (*P* < 0.05), but, contrarily, the percentage of ILT2^+^ NK cells was significantly increased in those patients (Figures 2A,B). Further, patients harboring del(11q) and trisomy 12, which have been associated with a poor clinical outcome in CLL (2–4), showed a significantly higher percentage of ILT2^+^ NK cells (*P* < 0.05; Figures 2C,D). Similarly, the percentage of ILT2^+^ NK cells was lower in patients with chromosome del(13q), which is associated with more favorable clinical outcome (*P* < 0.05) (5) (Figure 2E). No significant differences were observed in patients stratified by the presence of del(17p) (Figure 2F).

Altogether, these results indicate that the expression of the inhibitory molecule ILT2 is decreased on leukemic cells of CLL patients, but it is increased on NK cells of CLL patients, particularly in those with bad prognostic features.

### Lenalidomide Modulates the Expression of ILT2 and its Ligands in CLL Patients

**Figure 3:**
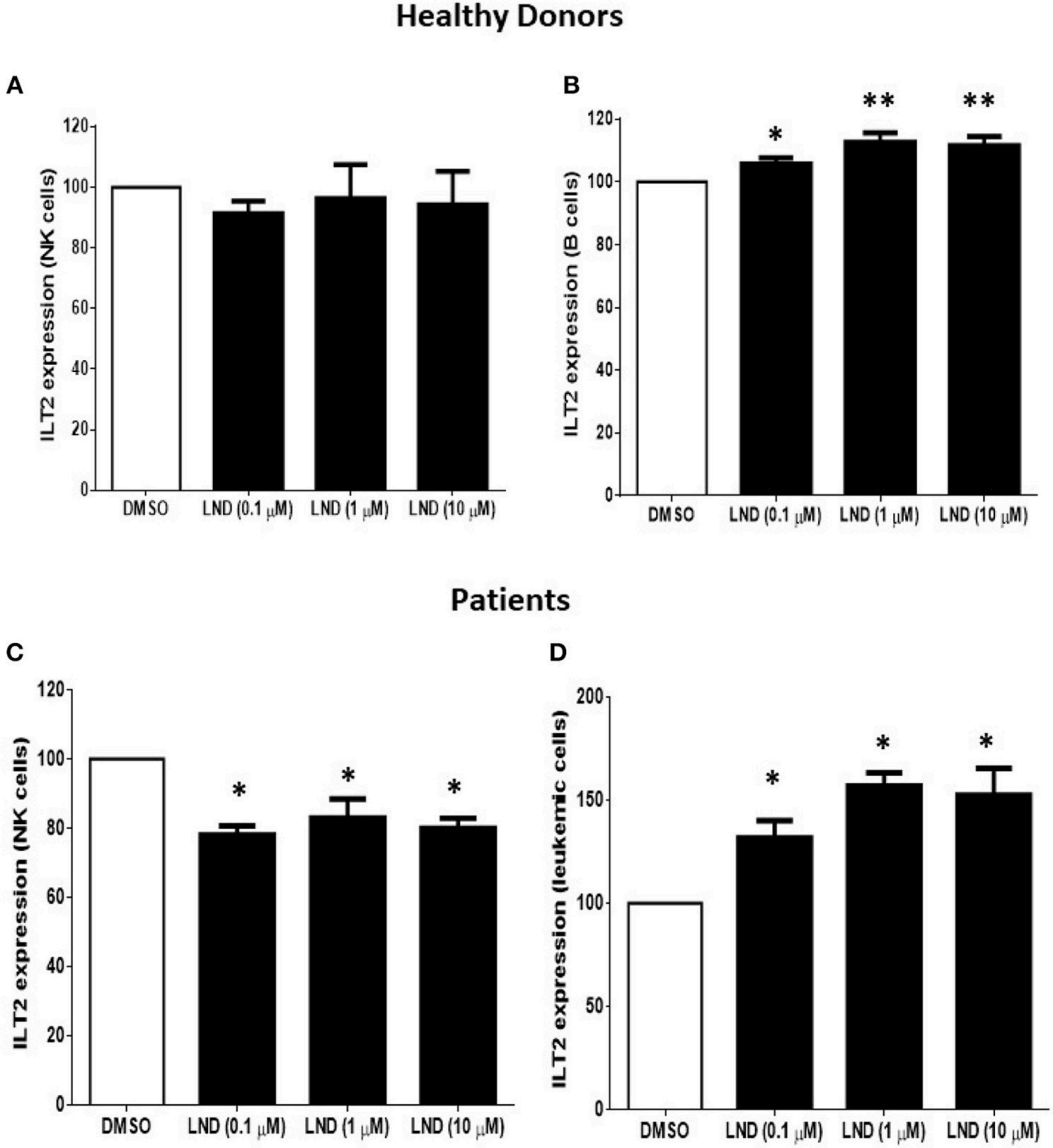
Effect of lenalidomide on ILT2 expression on NK cells and B cells from healthy donors and patients with CLL. The expression of ILT2 on NK cells and B cells from healthy donors **(A,B)** and patients with CLL **(C,D)** was evaluated by flow cytometry in PBMCs obtained from 4 CLL patients and 6 controls after the treatment with different doses of lenalidomide (LND) (0.1, 1, and 10 μM) for 7 days. The figure shows the comparison of the MFI of ILT2 expression normalized to the DMSO condition ± SEM. SEM, Standard Error of the Mean; Wilcoxon Matched-Pairs Signed Ranks test; ^*^P < 0.05, ^**^P < 0.01.

We next analyzed whether the immunomodulatory drug lenalidomide modulates the expression of ILT2. For this purpose, PBMCs from 4 patients with CLL and 6 healthy donors were incubated with increasing doses of lenalidomide (0.1 to 10 μM) for 7 days and the expression of ILT2 was evaluated on NK cells and B cells by flow cytometry. As shown in Figure 3, lenalidomide significantly decreased the expression of ILT2 on NK cells from CLL patients, with no marked effect observed in NK cells from healthy donors. Noteworthy, treatment with lenalidomide significantly enhanced ILT2 expression on the surface of both healthy and leukemic B cells, being this effect more potent in the latter.

**Figure 4:**
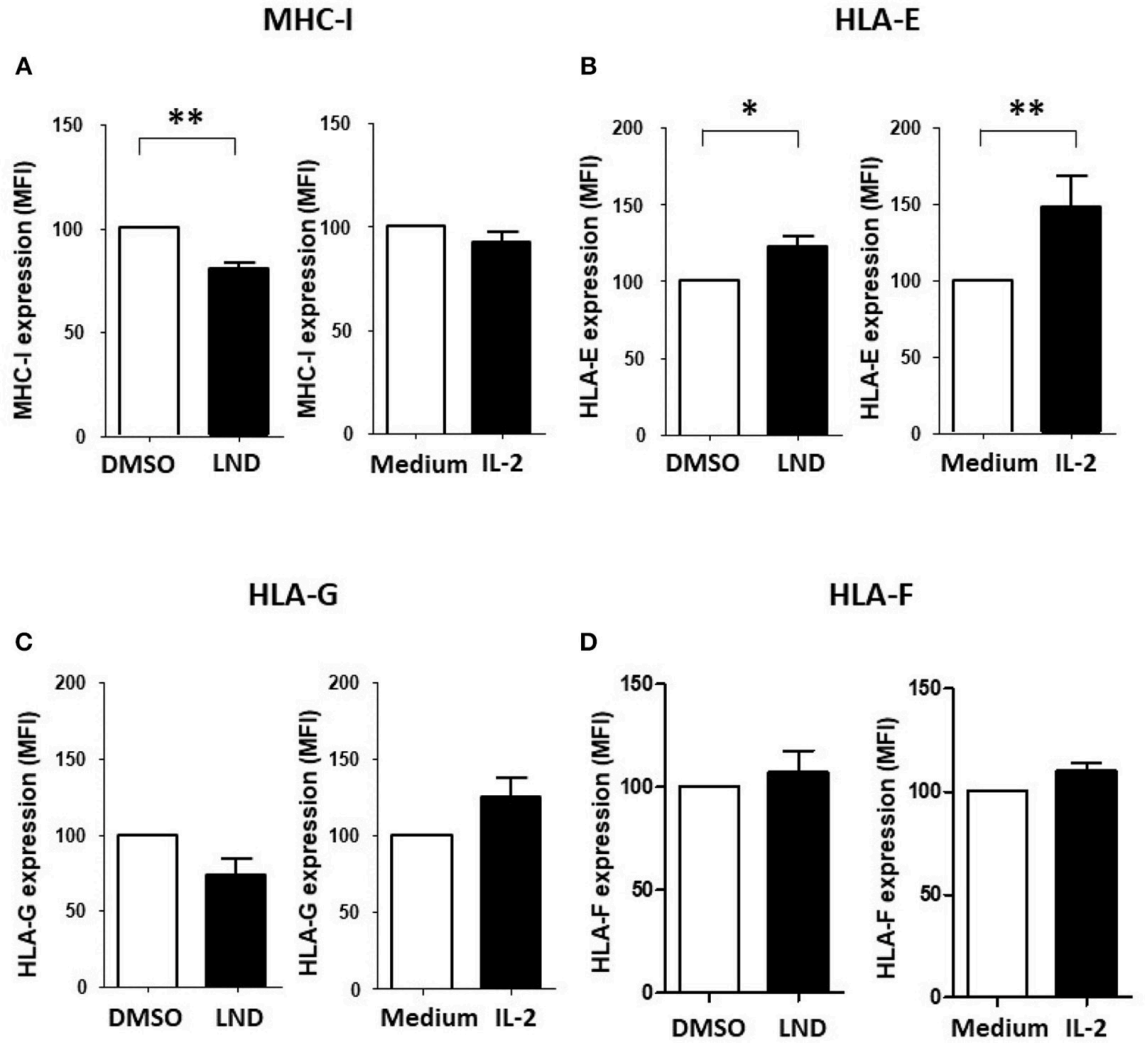
Effect of lenalidomide and IL-2 on the expression of ILT2 ligands on leukemic cells. The effect of lenalidomide (LND; 1 μM) and IL-2 (50 U/mL) on the expression of classical and non-classical MHC class I molecules on leukemic cells was analyzed by flow cytometry in PBMCs obtained from 10 CLL patients. The figure shows the comparison of the expression of MHC class I **(A)**, HLA-E **(B)**, HLA-G **(C)**, and HLA-F **(D)** on leukemic cells treated with both drugs. SEM, Standard Error of the Mean; Wilcoxon Matched-Pairs Signed Ranks test; ^*^P < 0.05, ^**^P < 0.01.

We reported that ILT2 ligand expression is deeply dysregulated on leukemic cells from patients with CLL (35). Specifically, the expression of HLA-E, HLA-F and HLA-G is diminished, while classical HLA class I molecules are increased on leukemic cells (35). Thus, we next asked whether lenalidomide modulates ILT2 ligand expression in CLL. As shown in Figure 4, lenalidomide reduced the levels of classical MHC class I molecules (*P* < 0.01) and increased HLA-E expression (*P* < 0.05) on leukemic cells; however, no significant effect was observed on the expression of HLA-G and HLA-F. Similarly, IL-2 increased the expression of HLA-E in these patients (*P* < 0.01).

Overall, these data indicate that lenalidomide partially restores the normal expression of ILT2 and its ligands in CLL.

### Effect of ILT2 Blockade and lenalidomide on CD69 Expression and Proliferation of NK Cells

**Figure 5:**
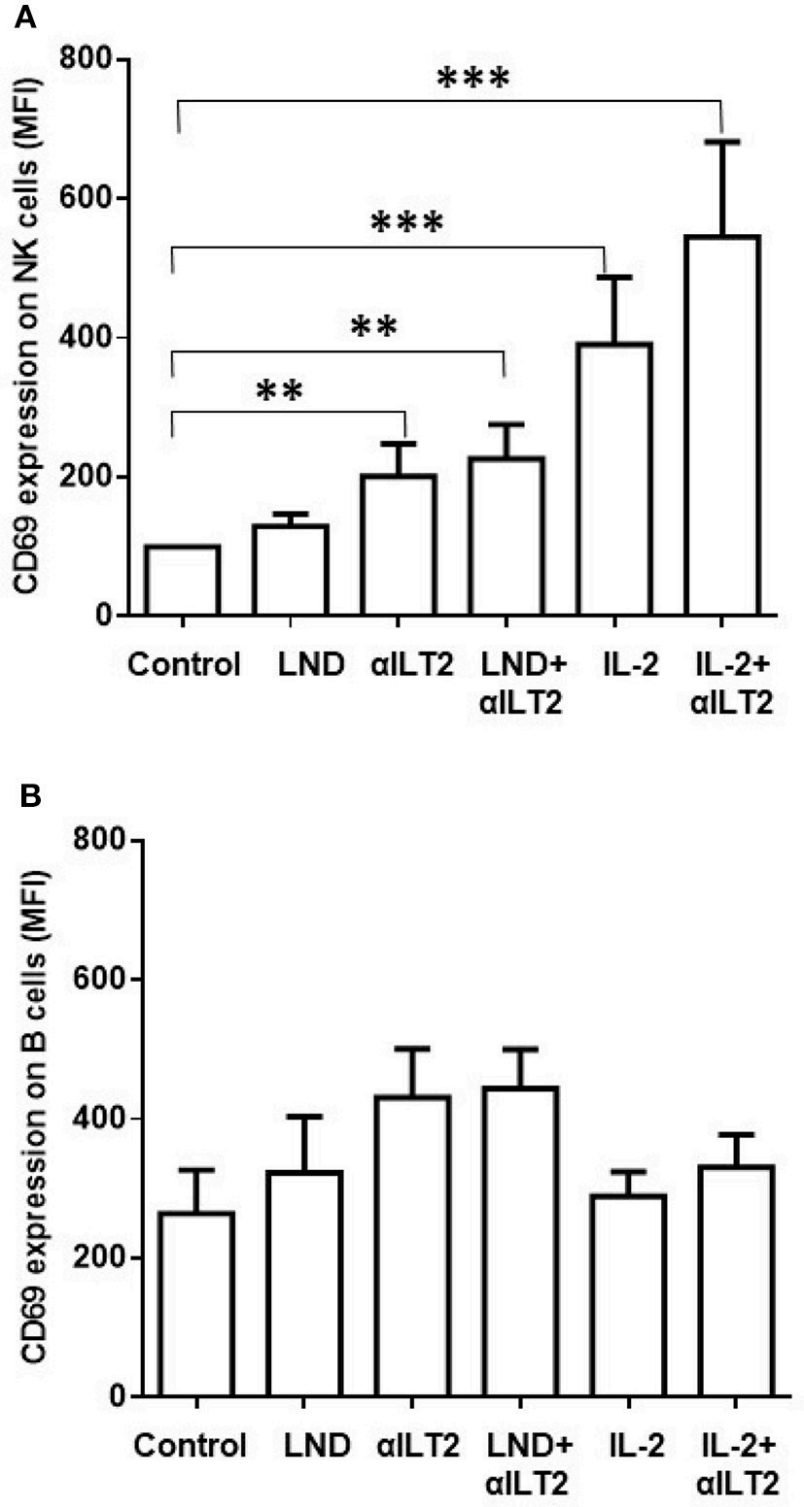
Effect of ILT2 blockade and lenalidomide on the activation of NK cells in CLL. Flow cytometry analyses were conducted to evaluate NK cell expression of CD69 in PBMCs obtained from 14 CLL patients and stimulated with anti-ILT2 blocking antibody (10 μg/ml) or irrelevant IgG_1_ (10 μg/ml) and lenalidomide (LND, 1 μM) or IL-2 (50 U/ml) for 7 days. The figure shows the comparison between the surface expression of CD69 detected on NK cells **(A)** and leukemic cells **(B)**. Bars represent the MFI of CD69 ± SEM for each condition. SEM, Standard Error of the Mean; Wilcoxon Matched-Pairs Signed Ranks test; ^**^P < 0.01, ^***^P < 0.001.

We next analyzed whether the HP-F1 antibody, which blocks the interaction between ILT2 and its ligands (36, 37), may increase the activation of NK cells. We also analyzed whether lenalidomide or IL-2 may cooperate with ILT2 blockade in this effect. Thus, PBMCs from 8 CLL patients were cultured in the presence of lenalidomide or IL-2 and the anti-ILT2 blocking antibody for 7 days and the expression of the immune activation marker CD69 was analyzed on NK cells by flow cytometry. ILT2 blockade and its combination with lenalidomide significantly increased the expression of CD69 on NK cells from CLL patients (Figure 5A). IL-2 also showed a cooperative effect with ILT2 blockade on NK cell activation. No significant effect of lenalidomide or anti-ILT2 blockade was observed on other NK cell activation and phenotypic markers including CD25, CD137, NKG2D or DNAM-1 (Figure 1S). Conversely, ILT2 blocking antibody and lenalidomide showed little capacity of activating leukemic cells (Figure 5B).

**Figure 6:**
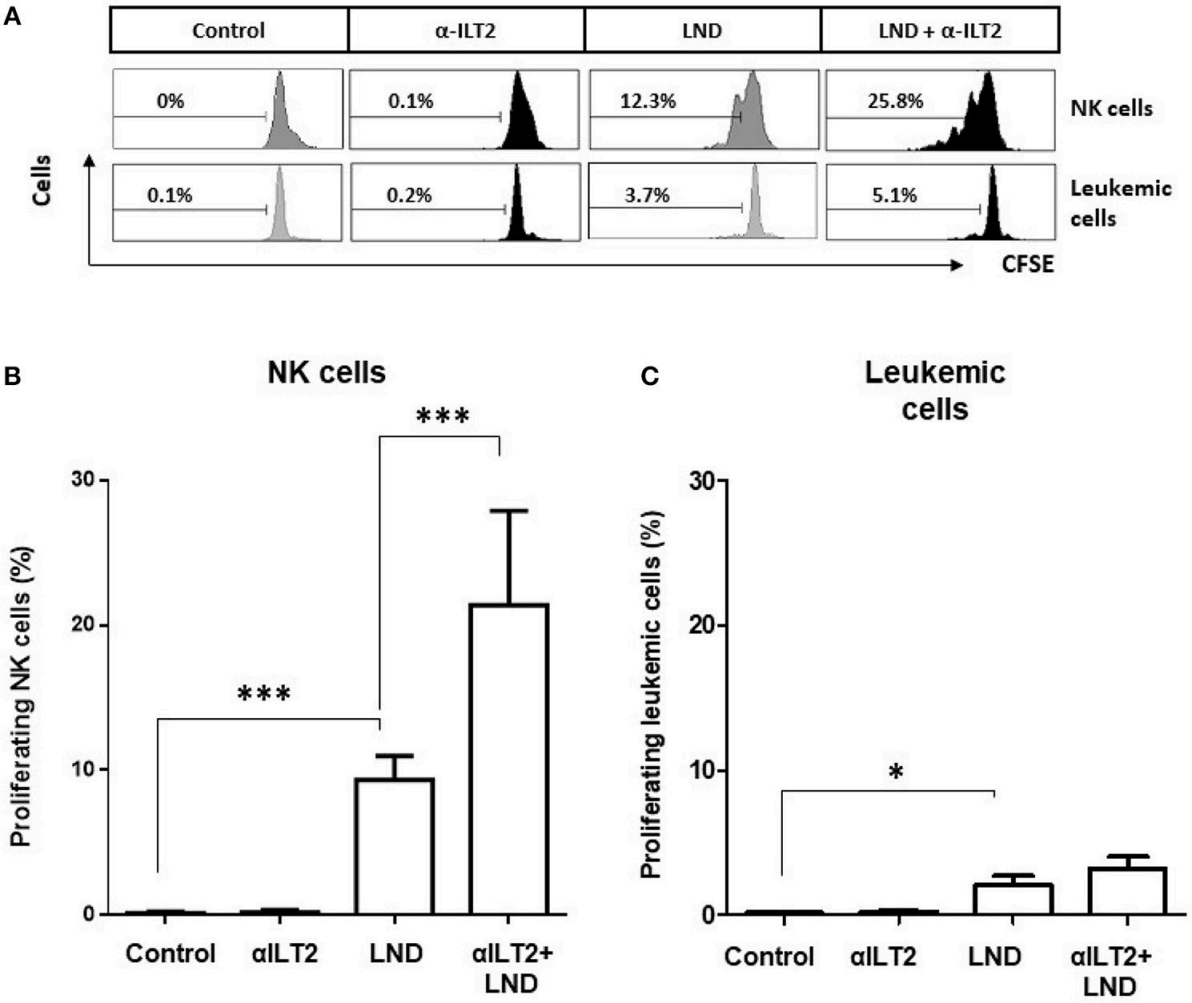
ILT2 blockade and lenalidomide promote NK cell proliferation in CLL. PBMCs from 11 CLL patients were stained with CFSE and cultured in the presence of anti-ILT2 blocking antibody (10 μg/ml) or irrelevant IgG_1_ and lenalidomide (LND, 1 μM) for 14 days. The proliferation of NK cells and leukemic cells was analyzed by evaluating the expression of CSFE by flow cytometry. **(A)** Histograms show the flow cytometry profiles corresponding to CFSE expression on NK cells (CD3^−^CD56^+^) and leukemic cells (CD19^+^ cells) of a representative CLL patient. **(B,C)** The histograms show the comparison of the percentage of proliferating NK cells **(B)** and leukemic cells **(C)** among the different experimental conditions analyzed. Bars represent the mean ± SEM from samples analyzed. SEM, Standard Error of the Mean; Wilcoxon Matched-Pairs Signed Ranks test; ^*^P < 0.05, ^***^P < 0.001.

The effect on NK cell proliferation was next analyzed. CFSE-stained PBMCs obtained from 11 CLL patients were treated with anti-ILT2 blocking antibody alone or in combination with lenalidomide for 14 days, and the effect on the proliferation of NK cells was determined by flow cytometry analysis (Figure 6A). Lenalidomide significantly increased the proliferation of NK cells (68-fold, *P* < 0.001) and this effect was strongly enhanced by ILT2 blockade (156-fold, *P* < 0.001; Figure 6B). As previously reported (32), lenalidomide increased in a lesser extent the proliferation of leukemic cells (*P* < 0.05), but this effect was not further enhanced by ILT2 blockade (Figure 6C).

### ILT2 Blockade and lenalidomide Promote the Elimination of Leukemic Cells

**Figure 7:**
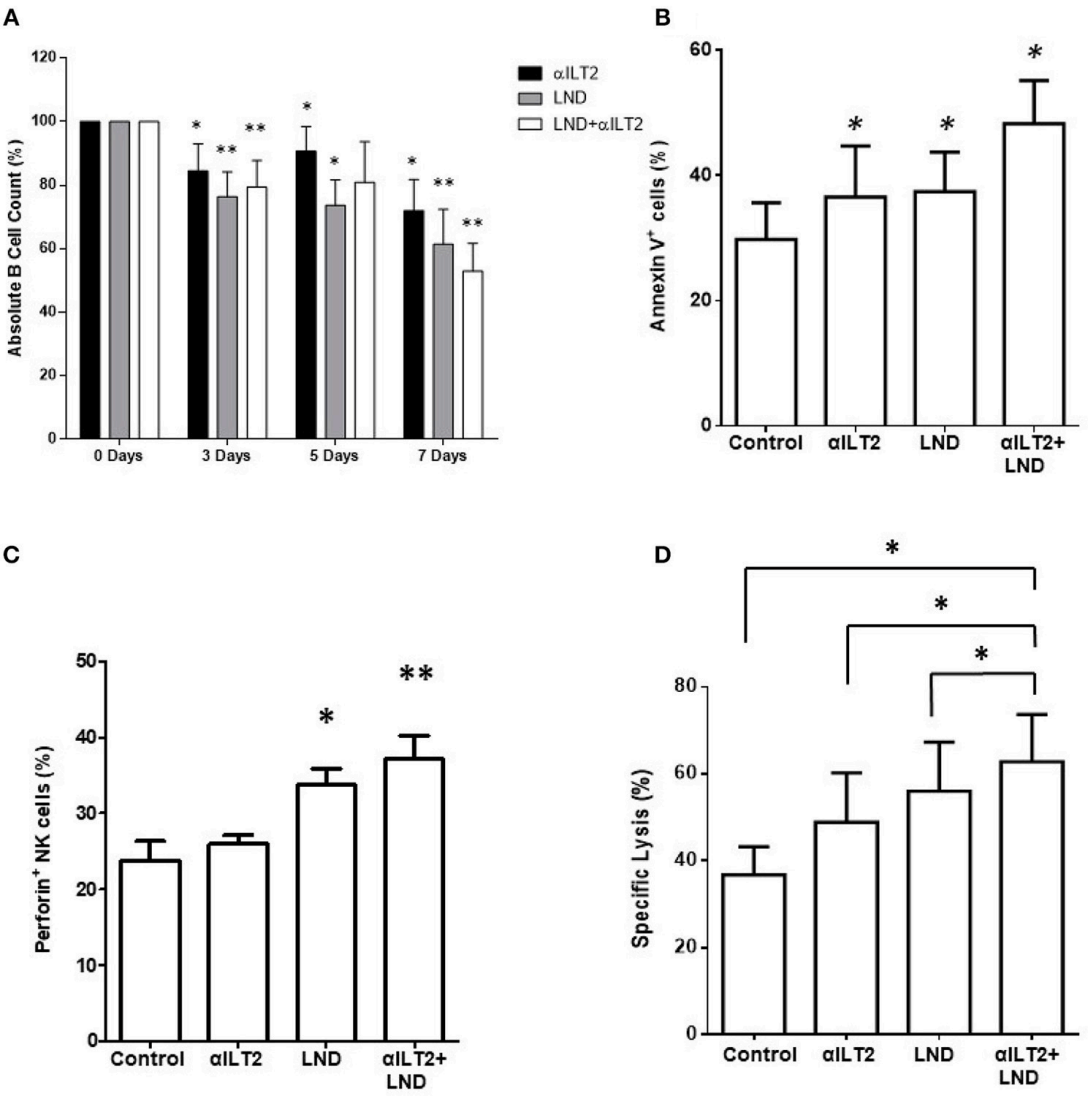
ILT2 blockade and lenalidomide promote NK cell cytotoxicity and the elimination of primary leukemic cells from patients with CLL. PBMCs obtained from 6 CLL patients were cultured in the presence of anti-ILT2 blocking antibody (10 μg/ml) or irrelevant IgG_1_ and lenalidomide (LND, 1 μM) for the time points indicated. **(A)** The absolute numbers of leukemic cells were then analyzed after 3, 5, and 7 days of treatment by staining with anti-CD19 antibody and PKH26 microbeads as a reference. **(B)** The effect of ILT2 blockade and lenalidomide (7 days) on the apoptosis of leukemic cells was evaluated by annexin V assay by flow cytometry (*n* = 11). **(C)** NK cell intracellular perforin expression was evaluated by flow cytometry analysis in PBMCs from 6 patients with CLL upon 7 days of treatment with anti-ILT2 blocking antibody (10 μg/ml) or irrelevant IgG_1_ and lenalidomide (LND, 1 μM). **(D)** NK cell cytotoxic activity against K562 leukemia cells after a week of treatment with anti-ILT2 blocking antibody (10 μg/ml) or irrelevant IgG_1_ and lenalidomide (LND, 1 μM) was evaluated by calcein assay (*n* = 5). Bars represent the mean ± SEM of the different treatments used. SEM, Standard Error of the Mean; Wilcoxon Matched-Pairs Signed Ranks test; ^*^P < 0.05, ^**^P < 0.01).

Next, the effect of ILT2 blockade and lenalidomide on the elimination of leukemic cells was evaluated. Thus, PBMCs from 6 patients with CLL were treated with anti-ILT2 blocking antibody and lenalidomide and the counts of leukemic cells were evaluated by flow cytometry at different time points (3, 5, and 7 days). Both ILT2-blockade and lenalidomide significantly diminished the numbers of leukemic cells in all the time points studied (Figure 7A). The observed reduction of leukemic cells was associated with an increase of leukemic cell apoptosis (Figure 7B). We showed that lenalidomide increases NK cell cytotoxic activity in CLL secondary to the production of IL-2 by CD4 T cells (32). In line with this, lenalidomide and ILT2 blockade significantly increased the intracellular levels of perforin on NK cells (Figure 7C). Concordantly, combination of lenalidomide and ILT2 blockade synergistically stimulated NK cell killing activity against K562 cells, as evaluated by calcein-based cytotoxic assay (Figure 7D).

Overall, our findings show that ILT2 blockade and lenalidomide have a significant effect on the elimination of leukemic cells of CLL patients, which was associated with an increased NK cell cytotoxic activity.

## Discussion

One of the key features of CLL is its association with a profound immunosuppression affecting to nearly all cells of the immune system including NK cells (9–11, 38). A relevant mechanism that account for the progressive loss of immune function in cancer is the sustained signaling of multiple inhibitory receptors, commonly known as checkpoints, such as PD1 and CTLA-4, which down-regulate the antitumor immune response (14, 39). These molecules have achieved increasing interest since targeting them has obtained an unexpected success in the treatment of some types of cancer. Likewise, the search for new checkpoint molecules in cancer is of crucial interest.

NK cells are cytotoxic lymphocytes specialized in early defense against virus infection and cancer cell transformation, particularly against hematological malignancies and metastasis (40). NK cell function is regulated by a delicate balance between signals provided by activating and inhibitory surface receptors that recognize their ligands on transformed cells (41–43). Negative regulation of NK cell function is controlled by an array of inhibitory receptors including killer immunoglobulin-like receptors (KIRs), NKG2A/CD94, the leukocyte immunoglobulin-like receptors, and the commonly considered checkpoint receptors (PD-1, TIM-3, LAG-3, and TIGIT). Interestingly, the loss of NK cell function in cancer is frequently caused by sustained signaling of multiple inhibitory receptors, and targeting these inhibitory receptors may restore NK cell activity providing a great benefit for these patients (15).

ILT2 is an inhibitory receptor involved in negative signaling in NK cells and other immune cells (16–18, 22, 36). Despite the fact that ILT2 has a significant capability of inhibiting the immune response, its role in cancer has achieved less attention than other checkpoint proteins. Nevertheless, we have recently shown that ILT2 is highly dysregulated in leukemic cells and CD8 and CD4 T cells of CLL patients, particularly in those patients having bad prognostic features, and it plays a significant role in the immunosuppression observed in those patients (35). Interestingly, targeting ILT2 restores T cell activity and ameliorates the immunosuppression. NK cells also exhibit reduced effector function in CLL, showing exhaustion features and functional defects in this disease (9–11). Our study shows that ILT2 may be involved in this immunosuppression observed in NK cells since it is overexpressed on NK cells of CLL patients, particularly in advanced patients and those showing bad prognostic features, and it is involved in the suppression of NK cell antitumor activity.

Lenalidomide (Revlimid; Celgene) is an immunomodulatory drug that is used as an antineoplastic agent in CLL and other hematological malignancies (30, 31, 44). In CLL, significant clinical responses, including molecular complete remissions in heavily pre-treated patients, have been observed (44). Lenalidomide does not induce direct apoptosis of leukemic CLL cells (25), but it regulates critical pro-survival cytokines and promotes the activation of T cells (28, 29). Moreover, it also increases NK cell proliferation, which correlates with clinical response (29–31), and augments NK cell-mediated cytotoxicity (33). We showed that lenalidomide induces the activation and proliferation of NK cells and enhances the natural cytotoxicity and ADCC activity of NK cells in CLL (32). We also showed that this effect in CLL is, at least in part, mediated by the production of IL-2 by CD4 T cells of CLL patients (32). Thus, lenalidomide is an interesting drug to be combined with therapeutic monoclonal antibodies since it may increase the antibody-dependent cytotoxicity of NK cells and it may cooperate with checkpoint inhibitors in the induction of the antitumor immune response mediated by NK and T cells. Consequently, an intense clinical and experimental research is been carried out to analyze new therapeutic strategies combining immunomodulatory drugs and monoclonal antibodies.

In this context, we decided to analyze the potential combination of lenalidomide and anti-ILT2 blocking antibody in the induction of the activity of NK cells of CLL patients. Our experiments show a novel effect of lenalidomide in regulating the immune response in CLL through the restoration, at least in part, of the expression of ILT2 and its ligands in leukemic cells of CLL patients. Thus, we previously showed that the expression of non-classical MHC class I molecules, namely HLA-G, HLA-E, and HLA-F, is decreased on leukemic cells (32). Contrarily, the expression of classical MHC class I molecules is increased in those cells (35). Here, our experiments show that lenalidomide increased the expression on leukemic cells of ILT2 and the non-classical MHC class I molecule HLA-E; but it decreased the expression of the classical ones. Similarly, lenalidomide also modulated the expression of ILT2 on leukemic cells; but, little effect was observed on NK cells, which suggests that lenalidomide may be an interesting drug to be combined with ILT2 blockade in the promotion of NK cell activity. In agreement, lenalidomide increased the activity, proliferation, and cytotoxicity of NK cells, and its effect was significantly potentiated by ILT2 blockade. These effects were associated, at least in part, with the induction of the apoptosis and elimination of leukemic cells. Despite the fact that the effect of lenalidomide and ILT2 blockade on T cells warrants further investigation, our results highlight their therapeutic potential in CLL.

It is of special interest the fact that a higher dysregulation of ILT2 expression on NK cells was observed in patients carrying a del(11q) and trisomy 12, which have been associated with enhanced clonal aggressiveness and worse evolution (2–6). The minimal region of deletion on 11q22.3–23.1 observed in CLL patients often involves the Radixin (RDX) and Ataxia telangiectasia mutated (ATM) genes. We reported that this deletion was also associated with a greater dysregulation of ILT2 expression in CD4 T cells and leukemic cells (35), which may suggest that those patients may have a greater immunosuppression, which could account for their poor clinical outcome. This also suggests that these patients, which showed poor response to chemotherapy treatment, may be more prone to response to new immunotherapy strategies. Nevertheless, additional specific studies in larger populations are needed in order to draw definite conclusions.

Overall, our results show that ILT2 signaling in NK cells is profoundly dysregulated in CLL and targeting ILT2 may be a potential therapeutic strategy to be explored in this disease. Furthermore, the effect of ILT2 blockade on NK cell activity may be potentiated by its combination with the immunomodulatory drug lenalidomide, which is currently being used in the treatment of this disease.

## Author Contributions

MV-Á collaborated in the design of the study, designed and performed the experiments, analyzed the data and collaborated with the manuscript edition. CS-B designed and performed the experiments and analyzed the data. SL-H performed experiments and analyzed the data. AG-R, AP, and EG-G diagnosed the patients, made the clinical evaluation and laboratory analyses of the patients, collected and analyzed the clinical data, and collaborated with the manuscript edition. MCV-Á participated in the analysis of patients and the data. AL-S participated in the experimental design and development of the study, and collaborated with the manuscript edition. SG designed the study, analyzed the data, and wrote and edited the manuscript.

### Conflict of Interest Statement

SG is the principal investigator of a research project in myeloma that is funded by Celgene. The remaining authors declare that the research was conducted in the absence of any commercial or financial relationships that could be construed as a potential conflict of interest.

## Supplementary Material

Figure 1S Effect of ILT2 blockade and lenalidomide on the modulation of activation markers on NK cells. The figure shows the comparison between the surface expression of CD25 and CD137 **(A)** or NKG2D and DNAM-1 **(B)** on NK cells (CD3^−^CD56^+^) from CLL patients (*n* = 4) after treatment with anti-ILT2 blocking antibody (10 μg/ml) or irrelevant IgG_1_ and lenalidomide (LND, 1 μM), IL-2 (50 U/ml) or the combination for 7 days. Bars represent the MFI ± SEM for each condition. SEM, Standard Error of the Mean; Wilcoxon Matched-Pairs Signed Ranks test; ^*^*P* < 0.05.
