# Supplementary figures and images for: Ig-Like Transcript 2 (ILT2) Blockade and Lenalidomide Restore NK Cell Function in Chronic Lymphocytic Leukemia
Source: Front Immunol. 2018 Dec 11;9:2917. doi: 10.3389/fimmu.2018.02917 (PMC6297751; doi:10.3389/fimmu.2018.02917)

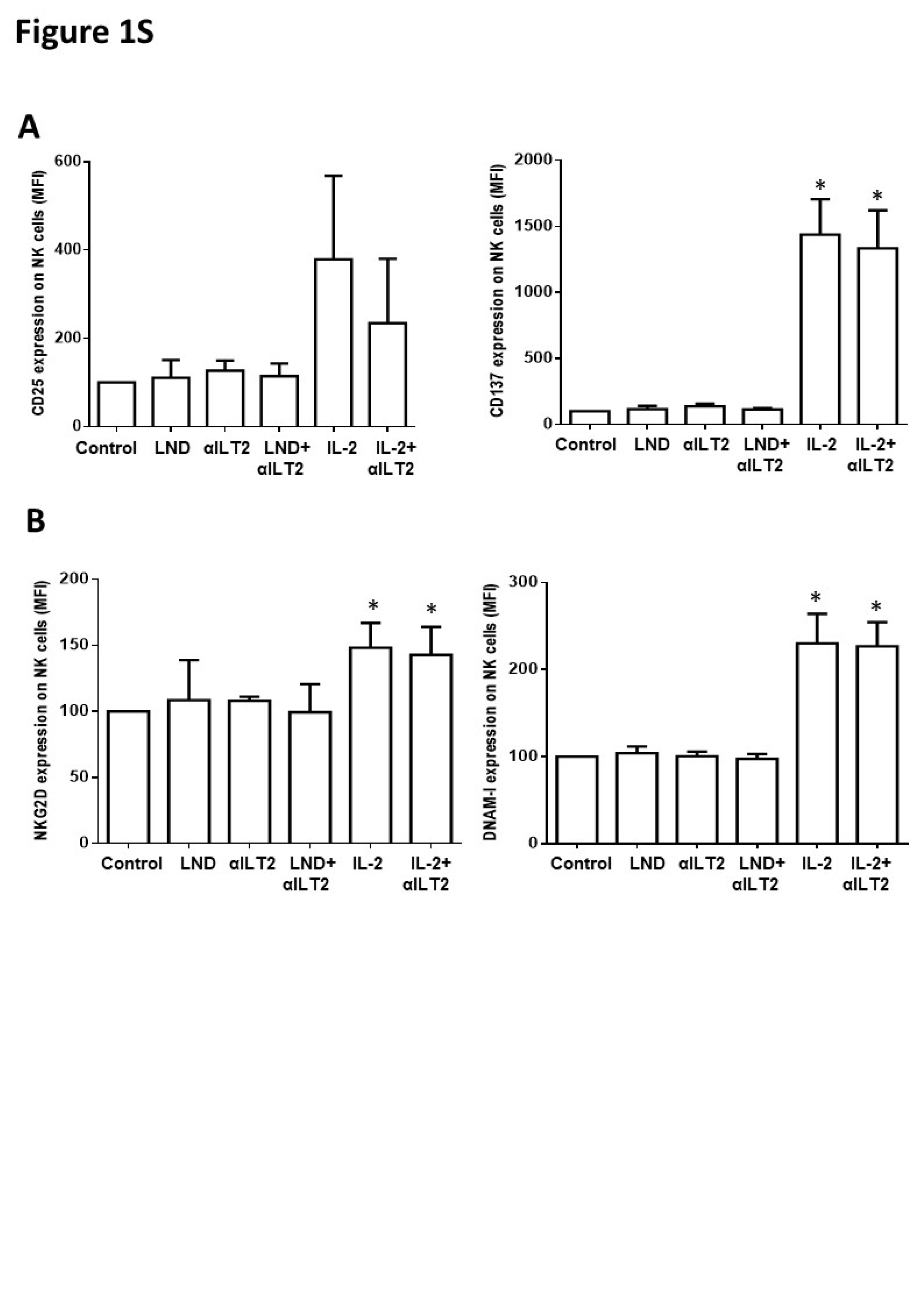

Supplement: Figure 1S — Effect of ILT2 blockade and lenalidomide on the modulation of activation markers on NK cells. The figure shows the comparison between the surface expression of CD25 and CD137 (A) or NKG2D and DNAM-1 (B) on NK cells (CD3−CD56+) from CLL patients (n = 4) after treatment with anti-ILT2 blocking antibody (10 μg/ml) or irrelevant IgG1 and lenalidomide (LND, 1 μM), IL-2 (50 U/ml) or the combination for 7 days. Bars represent the MFI ± SEM for each condition. SEM, Standard Error of the Mean; Wilcoxon Matched-Pairs Signed Ranks test; *P < 0.05. [file Image_1.tif]
